# Elevated level of the soluble receptor for advanced glycation end-products involved in sarcopenia: an observational study

**DOI:** 10.1186/s12877-021-02487-1

**Published:** 2021-10-07

**Authors:** Shou-En Wu, Yi-Lin Chiu, Tung-Wei Kao, Wei-Liang Chen

**Affiliations:** 1 Department of General Medicine, Tri-Service General Hospital, and School of Medicine, National Defense Medical Center, Taipei, Taiwan, Republic of China; 2 Department of Family and Community Medicine, Division of Geriatric Medicine, Tri-Service General Hospital, and School of Medicine, National Defense Medical Center, Number 325, Section 2, Chang-gong Rd, Nei-Hu District, 114 Taipei, Taiwan, Republic of China; 3 Department of Biochemistry , National Defense Medical Center, Taipei, Taiwan, Republic of China

**Keywords:** The soluble receptor for advanced glycation end-products, Sarcopenia, Muscle

## Abstract

**Background:**

The soluble receptor for advanced glycation end products (sRAGE) has been proposed to serve as a marker for disease severity, but its role in sarcopenia, an age-related progressive loss of muscle mass and function, remains elusive. This study examines the association between sRAGE and sarcopenia.

**Methods:**

A total of 314 community-dwelling elderly adults who had their health examination at Tri-Service General Hospital from 2017 to 2019 underwent protein analysis with enzyme-linked immunosorbent assay. The relationship with sarcopenia and its detailed information, including components and diagnosis status, were examined using linear and logistic regressions.

**Results:**

As for sarcopenia components, low muscle mass (β = 162.8, *p* = 0.012) and strength (β = 181.31, *p* = 0.011) were significantly correlated with sRAGE, but not low gait speed (*p* = 0.066). With regard to disease status, confirmed sarcopenia (β = 436.93, *p* < 0.001), but not probable (*p* = 0.448) or severe sarcopenia (*p* = 0.488), was significantly correlated with sRAGE. In addition, females revealed a stronger association with sRAGE level by showing significant correlations with low muscle mass (β = 221.72, *p* = 0.014) and low muscle strength (β = 208.68, *p* = 0.043).

**Conclusions:**

sRAGE level showed a positive association with sarcopenia, illustrating its involvement in the evolution of sarcopenia. This association is more evident in female groups, which may be attributed to the loss of protection from estrogen in postmenopausal women. Utilizing sRAGE level as a prospective marker for sarcopenia deserves further investigation in future studies.

**Supplementary Information:**

The online version contains supplementary material available at 10.1186/s12877-021-02487-1.

## Background

The receptor for advanced glycation end products (RAGE) is a multi-ligand receptor that was first reported in 1992 to interact with advanced glycation end products (AGEs), a cluster of non-enzymatically glycated and oxidized proteins and lipids, which have deleterious effects in both human and mice [1]. The soluble form of RAGE, known as sRAGE, lacks the transmembrane and cytoplasmic domains and circulates in blood and body fluids [2]. The role as an ally or enemy is conflicting. Some supported sRAGE as a protective factor according to the attenuation of AGE-RAGE axis and thereby preventing further disease progression after administration of recombinant sRAGE in animal studies [3–5]. Others reported higher levels of sRAGE were associated with increased incidence of cardiovascular events and mortality [6–8]. Studies targeting sRAGE are abundant in cardiovascular diseases and diabetes, but the role in sarcopenia, an aging-related muscle disorder, is less discussed. One of the multiple mechanisms of sarcopenia is attributed to chronic inflammation and accumulation of reactive oxygen species in muscle tissue [9, 10], which is exactly the known combat maneuver of AGEs, and the relationship within has been illustrated in previous literature [11, 12]. This raises our interest of whether sRAGE is also involved in the battle of sarcopenia. Our study provides a comprehensive investigation at both the gene expression level from a representative genetic database and at the protein level using enzyme-linked immunosorbent assay (ELISA).

## Methods

### Gene dataset

The Gene Expression Omnibus (GEO, https://www.ncbi.nlm.nih.gov/geo/) dataset is an internationally accessible repository of genomic data collected from different research units. It is constructed by the National Center for Biotechnology Information, aiming at provide all forms of high-throughput functional genomic data that can be freely downloaded.

### Study population for protein analysis (ELISA) of sRAGE

We enrolled 65-year-old and older community-dwelling elderly adults, who underwent a health examination at Tri-Service General Hospital (TSGH) from 2017 to 2019. This study has been approved by the institutional review board (IRB) of TSGH, and informed consents from participants were waived by the IRB with the assurance of anonymity and minimal risk throughout the analysis. Of the 335 total participants, we excluded those with insufficient necessary information for this study, including components of sarcopenia (skeletal muscle mass index(SMI), grip strength, and gait speed) and plasma levels of AGE and sRAGE. Ultimately, 314 participants were left eligible for further analysis, including 137 men and 177 women.

### Study design

**Fig. 1:**
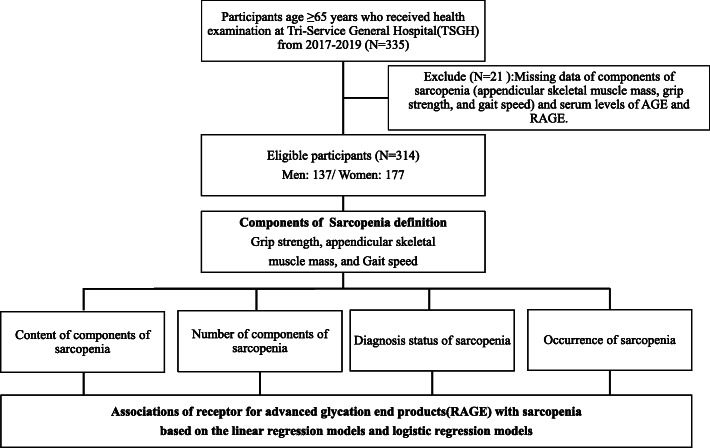
A flow diagram illustrating the research design. GEO: Gene Expression Omnibus; GSVA: Gene Set Variation Analysis; AGE: advanced glycation endproducts; RAGE: receptor for advanced glycation endproducts

**Fig. 2:**
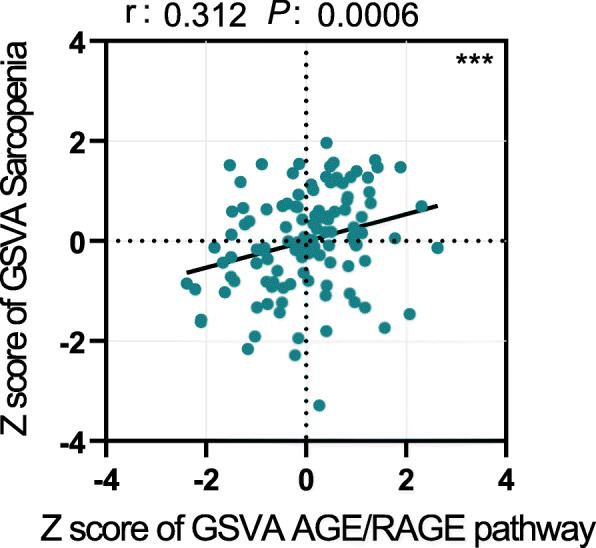
The gene set enrichment analysis of sarcopenia-associated gene and AGE/RAGE pathway-asssociated gene in GSE18732 from GEO database. Scatter plot showing the correlation between Z-score of GSVA sarcopenia and GSVA AGE/RAGE pathway. Pearson’s correlation coefficient(r) revealed a significant positive association. GEO: Gene Expression Omnibus; GSVA: Gene Set Variation Analysis; AGE: advanced glycation endproducts; RAGE: receptor for advanced glycation endproducts

This study could be separated into two main parts (Fig. 1). First, we investigated the GEO database for relevant data, expecting to prove the association between RAGE expression and sarcopenia. We then acquired the needed information from an additional database GSE18732, which was initially for another study. From this database, we searched for AGE/RAGE pathway-related and sarcopenia-related genes defined from Wikipath (https://www.wikipathways.org) and DisGeNet (http://www.disgenet.org), respectively [13]. The gene-set “AGE/RAGE pathway” consists of 79 genes that constitute the AGE/RAGE signaling (for a detailed gene list, please search WP2324 in Wikipath). The Sarcopenia gene-set was obtained from the “Enrichr” by searching for “sarcopenia” in “Term search”, and the original source is from the DisGeNet, a discovery platform which provided knowledge for genotype–phenotype relationships. The gene-set of “Sarcopenia” consists of 27 genes, most of which have been confirmed to be associated with sarcopenia (refer to the DisGeNet annotation). Subsequently, we calculated the GSVA (gene-set variation analysis) scores (details described in the following) in each group and then performed a linear regression to evaluate their relationship (Fig. 2) as well as the relationship between the mass of the arm, leg, and limb and the GSVA score of AGE/RAGE pathway (Additional Fig. 1).

Following the confirmation of the significant correlation from genetic data, we anticipated to verify certain results in clinical subjects by retrieving plasma samples from elderly participants’ health examinations in TSGH and analyzing their sRAGE levels using ELISA commercial kits. We then analyzed the association between sRAGE and sarcopenia (information collected from the items performed in the health examination), utilizing logistic and linear regression. Not only was the occurrence of sarcopenia evaluated (Additional Table 2), detailed sub-analyses were also performed, including components (Table 2 and Additional Table 1) and diagnosis status of sarcopenia (Table 3).

Double validation from gene and protein levels offered a more comprehensive view of this study.

### Gene expression analysis and GSVA

The resource of gene was obtained from the additional database GSE18732. This profile encompassed 118 participants’ vastus lateralis (a skeletal muscle) mRNA expression data. We obtained relevant clinical information for the present study, including demographic characteristics, medication, laboratory data, and arm, leg, and limb fat mass.

GSVA is a one of the gene-set enrichment methods that assesses the relative enrichment of a gene set in a nonparametric, unsupervised manner [14]. It produces a GSVA enrichment score for ranking and comparisons on the relevance of a target phenotype. In our study, we calculated the sarcopenia GSVA score according to a cluster of sarcopenia-associated genes defined from DisGeNet, a knowledge platform that contains the full spectrum of human diseases. On the other hand, we used the AGE/RAGE pathway-associated gene clusters provided by Wikipath to calculate the GSVA scores.

For GSVA calculation, we used the GSVA package in R and input the gene expression matrix file and gene-set file respectively, and performed the calculation under the default parameter settings, please refer to the Additional Table 3 for the gene list used.

### Elisa

ELISA was used for the quantitative determination of AGEs using a commercially available kit (Cusabio Biotech Co. Ltd., Wilmington, DE, USA) according to the manufacturer’s protocol. Inter- and intra-assay coefficients of variation were 12.5 and 8.2, respectively, for AGEs. sRAGE in plasmawere marked with the immunoenzymatic method (ELISA) using the Human Receptor for Advanced Glycation End Products (RAGE/AGER) ELISA Kit (Cusabio, Fannin St. Houston, TX, USA) by a sandwich method using anti-RAGE and anti-horseradish peroxidase antibodies. The whole procedure was performed in accordance with the manufacturers’ instructions.

### Measurement of components and definition of sarcopenia

We adopted the definition of sarcopenia from the 2019 consensus update of Asian Working Group for Sarcopenia [10]. There were three main components and three levels of diagnosis status depending on the components fulfilled; the three components were appendicular skeletal muscle mass (ASM), muscle strength, and physical performance, and they embraced several measuring methods, respectively. In the present study, we obtained ASM using bioelectrical impedance analysis, with the cutoff value of low ASM being < 7 kg/m^2^ for men and < 5.71 k/m^2^ for women. The low muscle strength that measured the handgrip strength had the cutoff values of < 28 kg for men and < 18 kg for women. Low physical performance was examined from low gait speed, which was defined as < 1 m/s for both genders. The three diagnoses were “possible,” “confirmed,” and “severe” sarcopenia. Possible sarcopenia was defined as having one component, being either low muscle strength or low physical performance; confirmed sarcopenia, having low muscle mass plus one of the other two components; and severe sarcopenia, having all three components.

### Covariates

Medical history recalls were obtained from self-reported questionnaires, which were involved in the health examination process, with questions like “Were you ever told by a doctor or diagnosed with certain disease?”

### Statistical analysis

The analytical software used in our study was SPSS (IBM Corp. Released 2013. IBM SPSS Statistics for Windows, Version 22.0. Armonk, NY: IBM Corp.). In Table 1, values in the continuous variables were expressed as mean and standard deviation, while values in the categorical variables were expressed in number and percentage (%). In the following tables, linear regression analysis was applied for comparison of continuous variables, whereas logistic regression analysis was performed for comparison of categorical variables. Pearson’s correlation coefficient (r) and odds ratio presented the strength of the association in the former and latter analyses, respectively. Two covariate adjustment models were presented: Model 1 = unadjusted; Model 2, the fully-adjusted model = adjusted for age, sex, smoking, comorbidities (hypertension, diabetes mellitus, myocardial infarction, angina, coronary artery disease, and chronic obstructive pulmonary disease). *P*-values < 0.05 indicate statistical significance.

**Table 1:** Characteristics of Study Population

Variables	Male(***n*** = 137)	Female(***n*** = 177)	***P*** value
**Continuous variable (mean** ± **SD)**^a^			
**Age (years)**	73.12 ± 8.41	71.82 ± 7.49	0.148
**Grip strength (kg)**	33.86 ± 8.05	20.23 ± 5.26	< 0.001
**SMI (ALM/ht**^**2**^**)(kg/m**^**2**^**)**	7.26 ± 0.79	5.79 ± 0.66	< 0.001
**Gait speed(m/s)**	1.13 ± 0.34	1.09 ± 0.30	0.330
**Number of comorbidity.**	0.77 ± 0.93	0.76 ± 1.02	0.922
**AGE(μg/mL)**	37.65 ± 17.80	37.48 ± 16.45	0.933
**RAGE(pg/ml)**	1204.24 ± 496.12	1280.28 ± 568.82	0.236
**Categorical variable (%)**^b^			
**HTN**	39.4%	31.1%	0.124
**DM**	10.9%	14.1%	0.403
**MI**	1.5%	0.6%	0.419
**Angina**	0.7%	1.1%	0.718
**CAD**	10.9%	5.1%	0.052
**COPD**	2.9%	5.6%	0.245

*Abbreviations*: *AGE* Advanced glycation end products, *sRAGE* soluble receptor for advanced glycation end-products, *SMI* skeletal muscle mass index, *HTN* hypertension, *DM* diabetes mellitus, *MI* myocardial infarction, *CAD* coronary artery disease, *COPD* chronic obstructive pulmonary disease

^a^Values in the continuous variables were expressed as mean and standard deviation

^b^Values in the categorical variables were expressed in number and percentage (%)

Pearson’s chi-square test was applied for comparison of categorical variables, whereas ANOVA (Analysis of Variance) was applied for comparison of continuous variables

## Results

### Characteristics of the study population

The study population included 137 (44%) male and 177 (56%) female participants, respectively (Table 1), with an average age of 73.12 ± 8.41 for men and 71.82 ± 7.49 for women (*p* = 0.148). Of the three sarcopenia components, women had significantly lower values in SMI(kg/m^2^)(5.79 ± 0.66 vs. 7.26 ± 0.79 in men, *p* < 0.001) and grip strength(kg)(20.23 ± 5.26 vs. 33.86 ± 8.05 in men, *p* < 0.001). Nonetheless, both components have different cutoff values for sarcopenia diagnosis in men and women; thus, they were not regarded as confounding factors. The levels of total AGEs(μg/ml) (37.65 ± 17.8 in men, and 37.48 ± 16.45 in women, *p* = 0.933) and RAGE(pg/ml) (1204.24 ± 496.12 in men and 1280.28 ± 568.82 in women, *p* = 0.236) did not reveal a gender difference.

### Association of sarcopenia GSVA score and the mRNA expression of AGE/RAGE pathway GSVA score

Figure 2 shows a significant correlation between sarcopenia GSVA score and the mRNA expression of AGE/RAGE pathway GSVA score (r = 0.312, p < 0.001).

### Association of the lean body mass of the arm, leg, and limb and the total mRNA expression of AGE/RAGE pathway GSVA score

Additional Fig. 1 reveals that the lean body mass of the arm (r = − 0.131, *p* = 0.1588), leg (r = − 0.181, *p* = 0.0499), and limb (r = − 0.170, *p* = 0.0660) and the total (r = − 0.138, *p* = 0.1371) were inversely related with the mRNA expression of AGE/RAGE pathway GSVA score. Among them, the leg was the only site with statistical significance.

### Relationship between sRAGE and components of sarcopenia in both genders

Table 2 shows that the sRAGE level was significantly correlated to low muscle mass (β = 162.8, *p* = 0.012) and low muscle strength (β = 181.31, *p* = 0.011) in the total population. Low gait speed was the only component of sarcopenia that did not reach statistical significance. As we further categorized our participants by gender, none of the sarcopenia components revealed a similar association in males, while low muscle mass (β = 221.72, *p* = 0.014) and low muscle strength (β = 208.68, *p* = 0.043) revealed an association with sRAGE level in females. In addition, the value of β coefficient was higher in the female population, demonstrating a stronger association in this group.

**Table 2:** Association between sarcopenia components and RAGE in gender difference using linear regression analysis

Gender	Variables	RAGE			
		Crude β coefficients (95% CI)	***P*** Value	Adjusted β coefficients (95% CI)	***P*** Value
**All**	**Low muscle mass**	162.80 (36.44, 289.16)	0.012	164.04 (36.02, 292.06)	0.012
	**Low muscle strength**	181.31 (28.90, 333.71)	0.020	216.71 (50.88, 382.54)	0.011
	**Low gait speed**	− 157.24 (−324.85, 10.38)	0.066	− 155.84 (− 331.54, 20.37)	0.083
**Male**	**Low muscle mass**	69.51 (− 112.77, 251.78)	0.452	27.51 (− 159.62, 214.64)	0.772
	**Low muscle strength**	61.68 (− 183.08, 306.44)	0.619	−38.42 (− 323.85, 247.02)	0.790
	**Low gait speed**	− 165.77 (− 392.22, 60.68)	0.150	− 264.37 (− 501.42, − 27.32)	0.029
**Female**	**Low muscle mass**	221.72 (45.51, 397.93)	0.014	239.47 (65.45, 413.48)	0.007
	**Low muscle strength**	208.68 (7.04, 410.31)	0.043	303.28 (99.41, 507.14)	0.004
	**Low gait speed**	− 142.96 (− 388.08, 102.16)	0.251	−71.93 (− 325.17, 181.32)	0.576

^a^Adjusted covariates: age, sex, smoking, comorbidities (hypertension, diabetes mellitus, myocardial infarction, angina, coronary artery disease, and chronic obstructive pulmonary disease)

Additional Table 1 shows the calculation of the sum of sarcopenia components. In the group of 0 and 1 components, no relationship was revealed, while in the group of 2 and 3 components, a relationship with sRAGE level in all populations (β = 175.35, *p* = 0.036) and females (β = 263.39, *p* = 0.023) was noted. As mentioned above, females also had stronger association.

### Relationship between sRAGE and diagnosis status of sarcopenia in both genders

Table 3 shows the association between sRAGE level and confirmed diagnosis of sarcopenia in all populations (β = 436.93, *p* < 0.001) and females (β = 478.78, p < 0.001). In accordance with the aforementioned findings, females also showed a stronger association. No similar relationship was shown in the probable and severe status of sarcopenia.

**Table 3:** Association between different status of sarcopenia, and RAGE in gender difference using linear regression analysis

Gender	Variables	RAGE			
		Crude β coefficients (95% CI)	***P*** Value	Adjusted β coefficients (95% CI)	***P*** Value
**All**	**Probable**	−95.63 (− 343.44, 152.18)	0.448	−76.05 (−333.16, 181.06)	0.561
	**Confirmed**	436.93 (222.05, 651.82)	< 0.001	449.72 (229.39, 670.05)	< 0.001
	**Severe**	94.77 (− 173.60, 363.14)	0.488	154.42 (− 132.99, 441.84)	0.291
**Male**	**Probable**	−266.70 (− 844.50, 311.11)	0.363	− 284.20 (− 866.80, 298.41)	0.336
	**Confirmed**	263.85 (−150.26, 677.95)	0.210	154.92 (−291.92, 601.77)	0.494
	**Severe**	62.51 (−280.07, 405.08)	0.719	−52.99 (− 434.77, 328.79)	0.784
**Female**	**Probable**	−75.45 (− 365.88, 214.97)	0.609	20.07 (− 276.95, 317.09)	0.894
	**Confirmed**	478.78 (215.11, 742.45)	< 0.001	498.61 (238.77, 758.45)	< 0.001
	**Severe**	142.14 (− 281.71, 565.99)	0.509	320.55 (− 113.07, 754.17)	0.146

^a^Adjusted covariates: age, sex, smoking, comorbidities (hypertension, diabetes mellitus, myocardial infarction, angina, coronary artery disease, and chronic obstructive pulmonary disease)

### Relationship between sRAGE and occurrence of sarcopenia in both genders

Additional Table 2 shows that elevated sRAGE level increased the risk of sarcopenia with odds ratio > 1 in all populations (OR = 1.001, *p* = 0.022) and females (OR = 1.001, p = 0.036).

## Discussion

Our study highlights the significant positive relationship between sRAGE and sarcopenia. First, genetic data from GEO database revealed a positive association between AGE/RAGE GSVA and sarcopenia GSVA scores. Secondly, ELISA demonstrated that sRAGE level and sarcopenia were associated from various perspectives, including the content of components, the sum of components, and different diagnosis statuses. Moreover, subgroup analysis revealed a gender difference as significant associations were exhibited in females but not in males.

Previous studies focusing on similar issues can be divided into two clusters: AGE with sarcopenia/muscle loss [11, 12] and RAGE with muscle diseases [15] but no robust evidence in sRAGE. In light of the precursory concepts, we speculated that sRAGE also possesses a relationship with sarcopenia and conducted our study accordingly. Our results not only confirmed our hypothesis but also offered several additional information worth discussing.

The underlying mechanisms of the interaction between AGE/RAGE and muscle have caught much of our attention. With respect to AGEs, a group of non-enzymatically glycated and oxidized proteins and lipids raised early awareness since the accumulation led to higher risk of muscle weakness in type 1 and 2 diabetes patients [11, 16] (referred as diabetic myopathy, a complication of diabetes) and healthy elderly population [12, 17]. These human studies mostly utilized skin autofluorescence, a noninvasive assessment method of AGEs, as a detection tool [18]. In vivo studies in mice [19] and in vitro studies [20] further suggested possible mechanisms, including impaired cellular signal transduction pathway in muscle (up−/down phosphorylation of different factors) [21]; cellular structure alteration by glycation and cross-linking, which increases muscle stiffness [22, 23]; and critical enzyme (e.g., creatine kinase) modification that originally contributes to the energy production of skeletal muscle [24]. Afterwards, these downstream consequences have been demonstrated to be largely attributed to the AGE/RAGE axis activation. RAGE, on the other hand, is better described as a mediator in muscle physiology, which should be expressed constitutively in muscle development and regeneration stages, but turns to lower levels in mature differentiated cells [25]. Aberrant overexpression of RAGE stimulated by several ligands triggers inflammation and oxidative stress, resulting to pathological conditions in muscle. For instance, increased levels of S-100B (one RAGE ligand) and RAGE are observed in experimental animal models of myasthenia gravis [26, 27]; and overexpression of HMGB1, CML(both RAGE ligands), and RAGE was found in the muscle fibers of polymyositis and dermatomyositis patients [28, 29]. As for sRAGE, a Korean study revealed the association between low levels of sRAGE and low muscle mass, suggesting a protective role of sRAGE against sarcopenia [30]. However, it was a cross-sectional study which could not draw causal inference. In contrast, a recent nested case-control study in four aging European cohorts concluded that sRAGE is a risk biomarker for frailty [31]. Another cohort study demonstrated sRAGE is an independent predictor of mortality among frail individuals [7]. These results seem conflicting, especially when reckoning with the role of sRAGE as a decoy receptor for membrane-bound RAGE, which binds RAGE ligands but does not initiate the AGE/RAGE axis and hence neutralizes the deleterious impacts [32]. Nevertheless, an innovative view argued that sRAGE levels may not be sufficient enough to antagonize RAGE-ligand interactions in light of the inflammatory situations where RAGE itself is also upregulated [33]. On the other hand, the elevated sRAGE level may be attributed to the overstimulation of cell surface RAGE by inflammatory signals, and thus increases the ectodomain shedding into circulation [34]. Some scientists therefore embrace the viewpoint that sRAGE is a prospective biomarker of disease but the clinical applications should be distinguished depending on different scenarios [2, 33]. Findings in the present study proposed that sRAGE may incline to a be a predictor of risk in sarcopenia.

Some extended findings in this study aroused our interest. First, the sub-analysis, which grouped participants by gender, revealed a significant association in females but not males. The gender-specific difference in AGE/RAGE signaling has been proposed in cardiovascular diseases [35] and metabolic syndrome [36]. A randomized controlled trial provided evidence that higher levels of AGEs and AGE/soluble RAGE (sRAGE) ratio were associated with lower physical functioning solely in women [37], which may be due to estrogen [36], since AGES, RAGE, and sRAGE levels were altered in postmenopausal women in hormone replacement therapy (HRT). Our study, which showed a strong connection between RAGE and saropenia in postmenopausal women (the average age of our female participant was 71.82), embraced the viewpoint that the loss of estrogen protection may pose a threat to muscle tissue through RAGE signaling pathway. On the other hand, considering that the evaluation of muscle strength in the current study adopted the measurement of handgrip strength, arthritis may be one interfering factor causing the decrease in strength. Arthritis displays a striking gender imbalance with female accounting for the majority of cases [38]. Furthermore, AGE/RAGE axis has been demonstrated to be involved in the pathogenesis of arthritis [39, 40], while sRAGE levels revealed association with the inflammatory status of rheumatoid arthritis [41]. Given these points, it seems the stronger association with sRAGE in females in our study should take account of the effect by arthritis. Nevertheless, we surveyed our participants and none of them had history of arthritis. Overall, the female predominant association requires further studies to provide more conclusive evidence and possible mechanisms.

Secondly, significant associations were present in certain subgroups but absent in others. Table 2 shows low muscle mass and strength significance but no significance for low gait speed. Table 3 confirms the significance of sarcopenia, but no significance for probable and severe sarcopenia. These minor differences suggest a stepwise evolution in sarcopenia. Two of the latest internationally accepted diagnostic criteria of sarcopenia applauded this concept. The European Working Group on Sarcopenia in Older People 2, just revised in 2019, suggested an algorithm that distinguished the diagnosis status into three groups [9]. The diagnosis of “probable sarcopenia” is made by confirming low muscle strength; “confirmed sarcopenia,” low muscle quantity (muscle mass); and “severe sarcopenia,” low physical performance (e.g., gait speed). The Asian Working Group for Sarcopenia, latest update published in 2020, also proposed an algorithm which defined “sarcopenia” as having low muscle mass plus one of the other two components and “severe sarcopenia” as having all three components [10]. Our results illustrated that the community-dwelling population in our study followed regular aging process, suffering from a decline in muscle mass and strength, but could still maintain a relatively healthy physical performance, which conformed to the aging process of muscle proposed by geriatric committees and experts.

This research has several limitations. First, AGEs are heterogeneous molecules and therefore the detection lacks a standardized method [42]. Different methodologies have their strengths and weaknesses. For plasma samples, liquid chromatography-mass spectrometry and ultra-high performance liquid chromatography both provide highly sensitive results with good resolution, but are more expensive and requires trained personnel. ELISA has the merit of simplicity and speed, but lacks enough antibody specificity and suffers from interference with glycation-free adducts. On the other hand, sRAGE is mainly measured by ELISA. Given that our main target was sRAGE, we utilized ELISA for both AGE and sRAGE detection, but further studies with mass spectrometry is warranted for valid and accurate results. Secondly, this is a cross-sectional study in which exposure and outcome were measured simultaneously; therefore, the casual relationship cannot be clarified. In addition, information on risk factors that may influence the outcome (e.g., medical history and health conditions) was obtained from self-reported questionnaires, and thus possible recalling biases may exist. Thirdly, the study population was confined to Taiwanese community-dwelling elderly adults from a single hospital. Results may vary in different ethnic groups and age distribution, warranting further studies to examine whether similar relationships apply to the above groups.

## Conclusions

sRAGE level is positively associated with sarcopenia, illustrating that sRAGE may be a perspective marker in age-related muscle change. This association showed female predominance in our study, which may be attributed the modulation by estrogen but requires further studies to get better understanding. The detection of sRAGE for sarcopenia in clinical practice deserves investigation in future work.

## Supplementary Information

**Additional file 1: Figure S1.** Scatter plot showing the correlation between Z-score of lean mass of arm, leg, and limb and GSVA AGE/RAGE pathway. **Table S1.** Association between numbers of sarcopenia component and RAGE in gender difference using linear regression analysis. **Table S2.** Association between RAGE and the occurrence of sarcopenia in gender difference using logistic regression analysis. **Table S3.** Gene list table for GSVA score calculation.

## Data Availability

There are 2 database that was used in the work. The first database is available on The Gene Expression Omnibus (GEO, https://www.ncbi.nlm.nih.gov/geo/). The second database contains single-institute data of TSGH and are within the manuscript and its Supporting Information files.

## Abbreviations

AGEs: Advanced glycation end products
RAGE: Receptor for advanced glycation end products
sRAGE: Soluble receptor for advanced glycation end products
GSVA: Gene-set variation analysis
HMGB1: High mobility group box1
ELISA: Enzyme-linked immunosorbent assay
SMI: Skeletal muscle mass index
ASM: Appendicular skeletal muscle mass
GEO: Gene Expression Omnibus
HRT: Hormone replacement therapy

## Declarations

Ethics approval and consent to participate: The study was performed under the guidelines of the Helsinki Declaration and approved by the Human Subjects Protection Offices (IRB) of the TSGH, National Defense Medical Center, Taiwan. Informed consents were collected from all participants before enrollment. This study does not include animal data.
