# Supplementary material for: Elevated level of the soluble receptor for advanced glycation end-products involved in sarcopenia: an observational study
Source: BMC Geriatr. 2021 Oct 7;21:531. doi: 10.1186/s12877-021-02487-1 (PMC8495916; doi:10.1186/s12877-021-02487-1)
Supplement: Supplementary file 1 — Additional file 1: Figure S1. Scatter plot showing the correlation between Z-score of lean mass of arm, leg, and limb and GSVA AGE/RAGE pathway. Table S1. Association between numbers of sarcopenia component and RAGE in gender difference using linear regression analysis. Table S2. Association between RAGE and the occurrence of sarcopenia in gender difference using logistic regression analysis. Table S3. Gene list table for GSVA score calculation. [file 12877_2021_2487_MOESM1_ESM.doc]

**LEGENDS FOR SUPPLEMENTARY MATERIALS**

**Additional Figure 1.** Scatter plot showing the correlation between Z-score of lean mass of arm, leg, and limb and GSVA AGE/RAGE pathway. Though most groups did not reach significance, pearson's correlation coefficients(r) revealed a negative trend of association.

GSVA: Gene Set Variation Analysis; AGE: advanced glycation endproducts; RAGE: receptor for advanced glycation endproducts.

**Additional Table 1.** Association between numbers of sarcopenia component, RAGE, and AGE in gender difference

**Additional Table 2.** Association between RAGE, AGE, and the occurrence of sarcopenia in gender difference.

**Additional table 3.** Gene list table for GSVA score calculation.

**Additional figure 1.** **Scatter plot showing the correlation between Z-score of lean mass of arm, leg, and limb and GSVA AGE/RAGE pathway.**


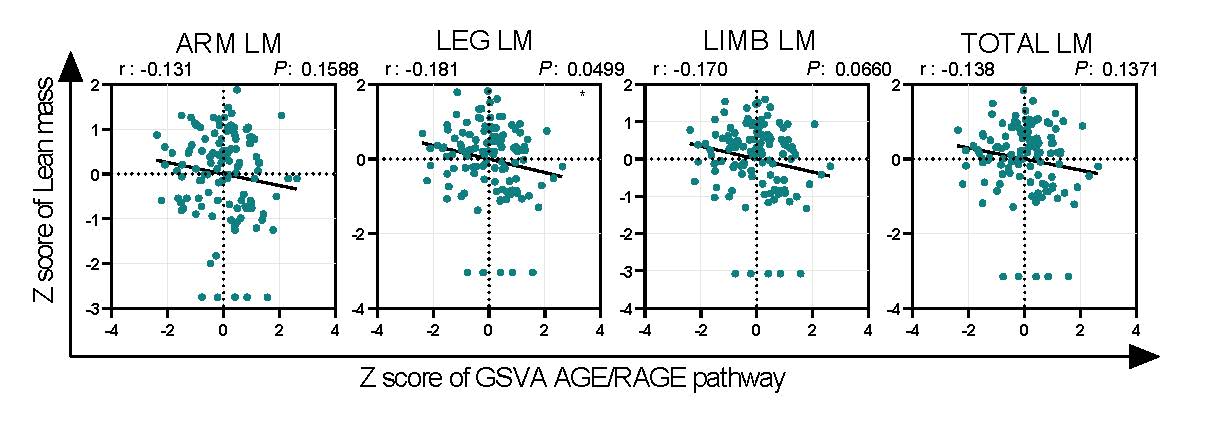


**Additional table 1. Association between numbers of sarcopenia component and RAGE in gender difference using linear regression analysis.**

| **Gender** | **Variables** | **RAGE** | | | |
| --- | --- | --- | --- | --- | --- |
|  |  | **Crude β coefficients**  **(95% CI)** | **P**  **Value** | **Adjusted β coefficients**  **(95% CI)** | **P**  **Value** |
|  | **0+1** | -22.38  -166.03, 121.28) | 0.759 | -20.45  (-165.13, 124.24) | 0.781 |
| **All** | **2+3** | 175.35  (11.65, 339.05) | 0.036 | 207.16  (31.55, 382.78) | 0.021 |
| **Male** | **0+1** | -88.93  (-294.81, 116.95) | 0.394 | -99.86  (-303.92, 104.19) | 0.334 |
|  | **2+3** | 25.21  (-217.52, 267.95) | 0.847 | -93.16  (-370.60, 184.28) | 0.507 |
| **Female** | **0+1** | 21.51  (-181.84, 224.86) | 0.835 | 50.99  (-148.44, 250.41) | 0.614 |
|  | **2+3** | 263.39  (36.74, 490.04) | 0.023 | 349.72  (122.57, 576.87) | 0.003 |

^a^ Adjusted covariates: age, sex, smoking, comorbidities (hypertension, diabetes mellitus, myocardial infarction, angina, coronary artery disease, chronic obstructive pulmonary disease, and arthritis).

**Additional table 2. Association between RAGE and the occurrence of sarcopenia in gender difference using logistic regression analysis.**

| **Gender** | **Variables** | **Sarcopenia** | | | |
| --- | --- | --- | --- | --- | --- |
|  |  | **Crude OR**  **(95% CI)** | **P**  **Value** | **Adjusted OR**  **(95% CI)** | **P**  **Value** |
| **All** | **RAGE** | 1.001 (1.000-1.001) | 0.022 | 1.001 (1.000-1.001) | 0.006 |
|  |  |  |  |  |  |
| **Male** | **RAGE** | 1.000 (0.999-1.001) | 0.555 | 1.000 (0.999-1.001) | 0.814 |
|  |  |  |  |  |  |
| **Female** | **RAGE** | 1.001 (1.000-1.001) | 0.036 | 1.001 (1.000-1.002) | 0.005 |
|  |  |  |  |  |  |

^a^ Adjusted covariates: age, sex, smoking, comorbidities (hypertension, diabetes mellitus, myocardial infarction, angina, coronary artery disease, chronic obstructive pulmonary disease, and arthritis).

**Additional table 3. Gene list table for GSVA score calculation.**

| **Gene-set** | **Gene symbol** |
| --- | --- |
| Sarcopenia | NR3C1, MSTN, HSD11B1, NFKB1, PRSS12, FOXO1, LMNA, MTOR, RPS6, TRIM63, MTHFR, LEP, GADD45A, ARNTL, NCAM1, MIP, SUPV3L1, IGF1, ACE, FBXO32, RUNX1, SOD1, TNF, ACTN3, GH1, REN, METTL21C |
| AGE/RAGE pathway WP2324 | STAT5A, PRKCD, PRKCB, STAT3, SRC, AKT1, PLA2G4A, STAT1, CASP8, IRS1, INHBB, ATF2, MMP9, NFKB1, FOXO1, SHC1, PRKCZ, JUN, CDC42, ALPL, DDOST, CASP9, NOS3, NCF1, RAC1, RAF1, CYCS, MAPK3, INSR, CASP3, IKBKB, MSR1, MMP2, TIRAP, SMAD3, MAP2K1, MMP13, MMP7, RHOA, MYD88, MSN, FOXO4, EGFR, STAT5B, PRKCA, MAPK9, NOS2, NFKBIA, HIF1A, MAPK8, LGALS3, MMP14, IRAK4, SP1, MAPK1, SOD1, EZR, MAPK14, AGER, ROCK1, SMAD2, JAK2, CHUK, RELA, DIAPH1, INS |
